# Genotyping strategy matters when analyzing hypervariable major histocompatibility complex‐Experience from a passerine bird

**DOI:** 10.1002/ece3.3757

**Published:** 2018-01-07

**Authors:** Silje L. Rekdal, Jarl Andreas Anmarkrud, Arild Johnsen, Jan T. Lifjeld

**Affiliations:** ^1^ Natural History Museum University of Oslo Oslo Norway

**Keywords:** bluethroat, Illumina MiSeq, Ion Torrent, *Luscinia svecica*, major histocompatibility complex

## Abstract

Genotyping of classical major histocompatibility complex (MHC) genes is challenging when they are hypervariable and occur in multiple copies. In this study, we used several different approaches to genotype the moderately variable MHC class I exon 3 (MHCIe3) and the highly polymorphic MHC class II exon 2 (MHCIIβe2) in the bluethroat (*Luscinia svecica*). Two family groups (eight individuals) were sequenced in replicates at both markers using Ion Torrent technology with both a single‐ and a dual‐indexed primer structure. Additionally, MHCIIβe2 was sequenced on Illumina MiSeq. Allele calling was conducted by modifications of the pipeline developed by Sommer et al. (BMC Genomics, 14, 2013, 542) and the software AmpliSAS. While the different genotyping strategies gave largely consistent results for MHCIe3, with a maximum of eight alleles per individual, MHCIIβe2 was remarkably complex with a maximum of 56 MHCIIβe2 alleles called for one individual. Each genotyping strategy detected on average 50%–82% of all MHCIIβe2 alleles per individual, but dropouts were largely allele‐specific and consistent within families for each strategy. The discrepancies among approaches indicate PCR biases caused by the platform‐specific primer tails. Further, AmpliSAS called fewer alleles than the modified Sommer pipeline. Our results demonstrate that allelic dropout is a significant problem when genotyping the hypervariable MHCIIβe2. As these genotyping errors are largely nonrandom and method‐specific, we caution against comparing genotypes across different genotyping strategies. Nevertheless, we conclude that high‐throughput approaches provide a major advance in the challenging task of genotyping hypervariable MHC loci, even though they may not reveal the complete allelic repertoire.

## 1 INTRODUCTION

The polymorphic and polygenic major histocompatibility complex (MHC) in many vertebrates is inherently difficult to genotype. The last decade has provided new sequencing platforms that may enable cheaper, faster, and more accurate and reproducible MHC genotyping (Babik, Taberlet, Ejsmond, & Radwan, 2009; Biedrzycka, Sebastian, Migalska, Westerdahl, & Radwan, 2017; Duke et al., 2015; Grogan, McGinnis, Sauther, Cuozzo, & Drea, 2016; Lighten, Oosterhout, & Bentzen, 2014). However, the task is still quite demanding in hypervariable study systems, partly due to the challenge of designing pipelines to separate true alleles from methodological artifacts (Babik, 2010; Grogan et al., 2016; Lighten et al., 2014; Sebastian, Herdegen, Migalska, & Radwan, 2016). Validation of true alleles through the use of replicate amplicons and pedigree information may however assist in quality control of the genotyping process of such systems (Gaigher et al., 2016; Grogan et al., 2016; Sommer, Courtiol, & Mazzoni, 2013; Zagalska‐Neubauer et al., 2010).

Major histocompatibility complex genes are crucial to trigger adaptive immune responses in jawed vertebrates and are among the most polymorphic genes known (Janeway, Travers, Walport, & Shlomchik, 2001). MHC class I (MHCI) genes encode transmembrane glycoproteins found on the surface of most cells. Peptides derived from intracellular pathogens bind specifically to the peptide‐binding region (PBR) of membrane‐bound MHCI molecules and are presented to CD8^+^ cytotoxic T cells. Similarly, peptides from extracellular pathogens are presented to CD4^+^ helper T cells by MHC class II (MHCII) molecules, which are located on specialized antigen‐presenting cells such as B cells, dendritic cells, and macrophages. Due to the specificity of the PBR, organisms with more MHC alleles are able to trigger an immune response against more pathogens. The polymorphism at MHC genes is believed to be influenced by several processes, including pathogen‐mediated balancing selection and sexual selection (see reviews by Edwards and Hedrick (1998) and Piertney and Oliver (2006)).

In Aves, the structure of the MHC varies immensely. While chicken (*Gallus gallus*) is described as having a “minimal essential MHC” (Kaufman et al., 1999), many non‐Galliform species exhibit an increased number of MHC loci (e.g., great snipe (*Gallinago media*; Ekblom, Grahn, & Höglund, 2003), blue petrel (*Halobaena caerulea*; Strandh, Lannefors, Bonadonna, & Westerdahl, 2011), and Eurasian coot (*Fulica atra*; Alcaide, Munoz, Martínez‐de la Puente, Soriguer, & Figuerola, 2014)). In Passeriformes, the MHC genes are extensively duplicated and highly diverse, and pseudogenes are commonly found (Westerdahl, 2007). For instance, Bollmer, Dunn, Freeman‐Gallant, and Whittingham (2012) detected a minimum of eight MHCI exon 3 (MHCIe3) and 23 MHCII β exon 2 (MHCIIβe2) loci in the common yellowthroat (*Geothlypis trichas*) using 454 sequencing, while Zagalska‐Neubauer et al. (2010) revealed numerous pseudogenes as well as at least nine transcribed MHCIIβe2 loci in collared flycatcher (*Ficedula albicollis*). Further, O'Connor, Strandh, Hasselquist, Nilsson, and Westerdahl (2016) described MHCIe3 diversity in 12 passerine species, in which the minimum number of loci ranged from four in the bluethroat (*Luscinia svecica*) to 19 in the willow warbler (*Phylloscopus trochilus*). High intra‐individual diversity was also found by Anmarkrud, Johnsen, Bachmann, and Lifjeld (2010), who used a traditional cloning and Sanger sequencing approach to identify 61 unique MHCIIβe2 alleles in 20 bluethroats and a minimum number of 11 functional loci.

For passerine birds, gene duplication and high‐sequence similarity at MHC loci due to gene conversion preclude single‐locus amplification when intron sequences are not known (Westerdahl, 2007). When performing PCR amplicon sequencing from multilocus gene targets, such as the MHC, several aspects may contribute to PCR‐induced biases. For example, similarity to primer sequence, GC content in primer‐binding sites and differences in secondary structures will influence the amplification success of the DNA template (Pawluczyk et al., 2015; Polz & Cavanaugh, 1998; Suzuki & Giovannoni, 1996). Hence, primer design is important in order to reduce PCR‐introduced biases. Many researchers now use “phusion primers” when performing amplicon sequencing. These are primer sequences with platform‐specific adapters, sample‐specific index tags (barcode), and other sequence motifs added to the target gene sequence. These motifs will generate a “primer tail”. This tail may thus introduce amplification biases if it has a noncompatible GC pattern to the nucleotides surrounding the primer‐binding motif, or if local secondary structures obstruct annealing of the primer.

Although Roche 454 pyrosequencing has been extensively applied in MHC studies on nonmodel organisms since 2009 (Babik et al., 2009), this platform is now being phased out and new technologies are applied. Of the available high‐throughput sequencing platforms, Ion Torrent semiconductor sequencing and Illumina MiSeq paired‐end sequencing are currently among the most appropriate alternatives for MHC genotyping due to read lengths and output. However, as for every sequencing method, these techniques are also subject to errors. In addition to substitution errors made by polymerases, chimera formation is common in multilocus PCR amplification (Kanagawa, 2003; Lenz & Becker, 2008). Further, homopolymer errors causing indels are abundant in Ion Torrent (Loman et al., 2012). Being aware of these pitfalls is essential, and it is important to take measures to minimize the impact of artifacts arising before or during sequencing. Thus, establishment of robust PCR approaches and allele‐calling pipelines is crucial for separating artifacts from true alleles.

Two main assumptions are generally made in the processes of recognizing artifacts in MHC studies: Artifacts should be less common than real alleles across and within individuals, and they should originate from true alleles (Babik et al., 2009). Based on these assumptions, Babik et al. (2009) used per individual frequencies to identify a threshold below which artifacts should occur. This was further elaborated by Galan, Guivier, Caraux, Charbonnel, and Cosson (2010) who established two thresholds: *T*_1_; the minimum number of reads *per sample* required for reliable genotyping, and *T*_2_; intra‐amplicon frequency corresponding to the minimum number of reads *per variant* to validate true alleles. However, as pure threshold approaches potentially misidentify alleles and artifacts (Lighten et al., 2014), stricter approaches are needed in complex MHC systems. In a MHCII study on flycatchers, Zagalska‐Neubauer et al. (2010) used a 2‐PCR‐3‐reads‐in‐each inclusion criteria, where the variants had to be present with at least three reads in two independent PCRs to be considered alleles. Further, in order to account for artifacts and allelic dropout, Sommer et al. (2013) established an expanded workflow for genotyping MHC in nonmodel organisms. Their allele‐calling pipeline relies on amplicon replicates, artifact detection, and relative intra‐amplicon frequencies after initial quality filtering of the sequencing reads. By combining this pipeline with a 1% threshold approach similar to Galan's *T*_2_, Grogan et al. (2016) genotyped MHC‐DRB in ring‐tailed lemurs (*Lemur catta*) across 454 and Ion Torrent platforms with consistent results. Introducing such a threshold of 0.4% also minimized the number of artifacts when genotyping the hypervariable MHCIe3 in sedge warblers (*Acrocephalus schoenobaenus*) using this pipeline (Biedrzycka et al., 2017).

Another MHC allele‐calling pipeline based on a stepwise threshold clustering methodology was developed by Stutz and Bolnick (2014). In this pipeline, sequences are clustered based on similarity and filtered, attempting to reveal artifacts and add their depths to the putative alleles from which they are originating. By incorporating platform‐specific error rates, Sebastian et al. (2016) have implemented this pipeline in the publicly available AmpliSAS tool.

Biedrzycka et al. (2017) compared four allele‐calling strategies for genotyping sedge warbler MHCIe3 including both AmpliSAS and the original workflow established by Sommer et al. (2013). They found high agreement (>90%) between these pipelines at coverages above 2,000 reads but argue for the use of coverages of >5,000 reads due to the increased reliability.

In our study, we further compare allele calling by modifying the Sommer pipeline and AmpliSAS by the use of family data, in order to facilitate genotyping of bluethroat MHC. We thus compare different aspects of genotyping strategies (i.e., sequencing approaches and allele‐calling pipelines) in a moderately variable MHC gene (MHCIe3) and a highly polymorphic MHC gene (MHCIIβe2) in the bluethroat, using two family sets (two offspring in each family, with their genetic parents). The MHCIe3 and MHCIIβe2 amplicons were sequenced on an Ion Personal Genome Machine™, applying two different primer tail approaches. Additionally, the MHCIIβe2 amplicons were sequenced on the Illumina MiSeq^®^ platform. Using these approaches, we aimed to test whether platform or sequence motif and length of the primer tail would bias the outcome. We modified the pipeline of Sommer et al. (2013) and the downstream analyses of the output from AmpliSAS software (Sebastian et al., 2016), and used allelic inheritance patterns between parent and offspring genotypes as additional support for the results. Accordingly, using family data, we wished to establish a workflow for robust genotyping of bluethroat MHCIe3 and MHCIIβe2, which is a premise for the use of these markers in ecological and evolutionary analyses.

## 2 MATERIALS AND METHODS

This study is based on DNA from blood samples of two offspring and their biological parents in two family groups of bluethroats (*L. svecica svecica*; Appendix S1). The eight individuals were sampled in the subalpine habitat of Øvre Heimdalen valley, Øystre Slidre, Norway (61°25′N, 8°52′E). Norwegian Animal Research Authority gave ethical permissions to the fieldwork (license 2014/53673 to AJ). Parentage was confirmed through a panel of microsatellites in another study (Sætre, Johnsen, Stensrud, & Cramer,unpublished data). DNA was extracted using E‐Z^®^ 96 Blood DNA Kit (Omega Bio‐Tek Inc. [D1199‐01]), following the protocol of the manufacturer.

### 2.1 Sequencing

All amplicons (for explanation of terms, see Appendix S2) were amplified in duplicates, with a unique barcode identifier for each replicate. Primer sequences and binding sites are provided in the Supplementary material (Appendices S3 and S4). In order to minimize PCR artifacts, the number of PCR cycles was reduced to 25 (Lenz & Becker, 2008). For detailed description of the amplification and sequencing, see Appendix S5.

**Figure 1:**
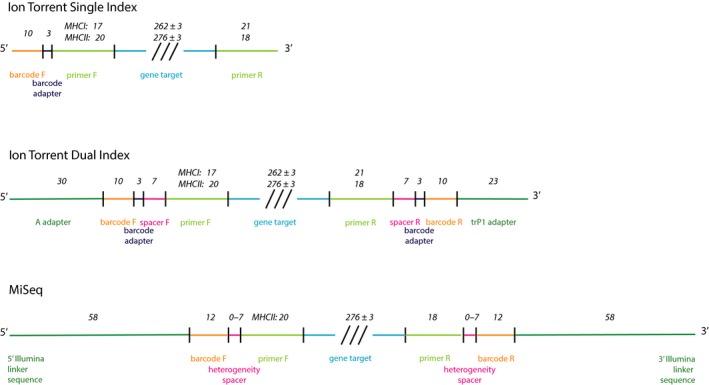
Primer setup used to amplify MHC class I exon 3 (MHCI) and MHC class II exon 2 (MHCII) in eight bluethroats. MHCI was sequenced in a single index run and in a dual index run on Ion Torrent. MHCII was additionally sequenced on Illumina MiSeq. The numbers are referring to the length of the respective parts of the primers

MHCIe3 was amplified using the primer pair MhcPasCI‐FW and MhcPasCI‐RV (Alcaide, Liu, & Edwards, 2013) and sequenced on an Ion PGM. Two primer structures were applied (see Figure 1); one in which barcode and barcode adapter were included only on the forward primer (single index; SI), and one including Ion Torrent adapter, barcode, barcode adapter, and seven nucleotide spacer motif on both forward and reverse primers (dual index; DI).

The primers MHCIIFihy‐E2CF and MHCIIFihy‐E2CR (Canal, Alcaide, Anmarkrud, & Potti, 2010) were used to amplify MHCIIβe2 by a similar SI and DI approach, and the amplicon sequencing was conducted on an Ion PGM. Additionally, MHCIIβe2 amplicons were generated by including Illumina Linker sequences, barcodes, and heterogeneity spacer motif (Fadrosh et al., 2014) on both forward and revers primers, and sequenced on Illumina MiSeq (see Figure 1).

### 2.2 Allele calling

Allele calling was conducted through two pipelines: one based on a previously published pipeline by Sommer et al. (2013), and one based on the software AmpliSAS (Sebastian et al., 2016).

#### 2.2.1 Modified pipeline from Sommer et al. (2013)

**Figure 2:**
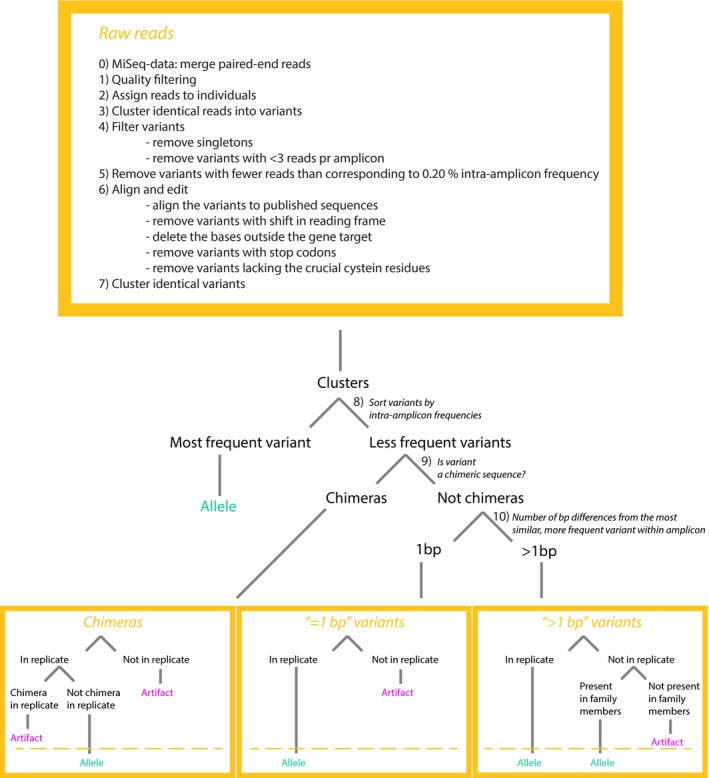
Flowchart over the pipeline modified from Sommer et al. (2013), conducted to genotype MHC class I exon 3 and MHC class II exon 2 in eight bluethroats

**Figure 3:**
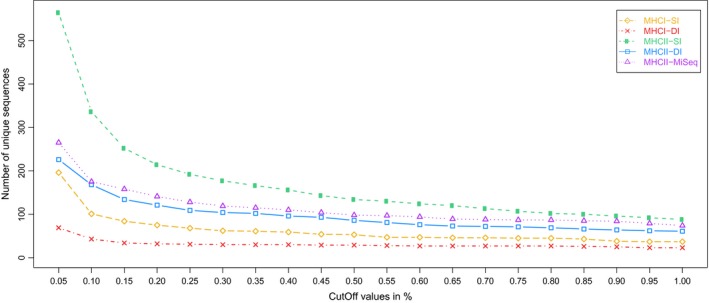
Number of unique sequences for different values of cut‐off, found within each strategy (MHC class, primer approach, and platform) in the eight bluethroat individuals. Only variants having three reads or more in any amplicon are included. Short and low‐quality reads are removed, but no further filtering was conducted before this step, in order to reveal any threshold where low‐frequency artifacts are likely included

A flowchart of this allele‐calling method is outlined in Figure 2, while a detailed description and comments on the modifications are provided in Appendix S6. In short, paired MiSeq reads were merged using FLASH (Magoč & Salzberg, 2011), and raw reads from all datasets were quality filtered using standard UNIX commands and fastx toolkit (http://hannonlab.cshl.edu/fastx_toolkit/). Barcode splitting and clustering of identical reads into variants were conducted by jMHC (Stuglik, Radwan, & Babik, 2011). Variants with less than three reads in any amplicon were discarded, as were amplicons with less than 500 reads in total. After this step, we established a threshold above which we expected to have included most true alleles and excluded most artifacts, based on the assumption that true alleles will amplify to a greater depth than artifacts will (Babik et al., 2009; Lighten et al., 2014). A cut‐off threshold of 0.2% was conservatively inferred from visually recognizing a change in number of unique variants included at different values of cut‐off (Figure 3). Hence, variants with intra‐amplicon frequency of less than 0.2% were discarded for the respective amplicons.

The remaining variants across the whole dataset were aligned to previously published sequences (GenBank accession number KU169737–KU169747 (MHCI; O'Connor et al., 2016) and HQ539575–HQ539614 (MHCII; Gohli et al., 2013)) with ClustalW (Thompson, Higgins, & Gibson, 1994) in MEGA7 (Kumar, Stecher, & Tamura, 2016), and trimmed correspondingly. Variants with shift in reading frame, stop codon, or lacking the conserved residues Cys7 and Cys70 (MHCI; O'Connor et al., 2016) or Cys10 and Cys75 (MHCII; Gohli et al., 2013) were discarded. Further, chimera detection was carried out using UCHIME (Edgar, Haas, Clemente, Quince, & Knight, 2011).

While the most frequent variant within each amplicon was scored as an allele, the remaining variants were divided into “=1 bp” and “>1 bp” variants, according to the number of base pair differences to their most similar, more frequent variant, found by MEGA7. Amplicon replicates were then utilized to score artifacts (chimeric variants scored as chimera also in replicate or not found within replicate above 0.2% threshold; “=1 bp” variants not found within replicate above 0.2% threshold; “>1 bp” variant not present above 0.2% threshold in any other amplicon from individuals within the same family group). The remaining variants within each amplicon were scored as alleles (see Figure 2).

For offspring with one failed amplicon, family information was used as a substitute for the replicate. Here, “=1 bp” variant or a chimeric variant was scored as an allele if present in parental genotypes. “>1 bp” variants were called as alleles if found in any other family member.

#### 2.2.2 AmpliSAS pipeline

As a second allele‐calling pipeline, we used the online tool suite AmpliSAT (Sebastian et al., 2016). After initial filtering (see Appendix S6), the datasets were explored in AmpliCHECK. Corresponding to the AmpliCHECK results, we set maximum number of alleles per amplicon to 60 for the algorithm implemented in AmpliSAS. Minimum amplicon depth was set to 500, while we used default platform‐specific error rates for substitutions and indels. In‐frame length was required for the dominant sequence within a cluster. Further, the frequency of the subdominant cluster with respect to the dominant frequency was changed from the default of 25% to 10%, in order to avoid clustering similar alleles with different amplification efficiencies (Biedrzycka et al., 2017). Lastly, variants with an intra‐amplicon frequency of less than 0.20% or with a depth below three reads were discarded. Variants of lengths exhibiting frameshifts in relation to the expected length, noncoding variants, and chimeras were also discarded.

Duplicates of all individuals enabled validation of alleles based on the presence in the replicate sample. Hence, only variants scored in both amplicon replicates of an individual were called as alleles. Error rates were calculated as the percentage of putative alleles not found in both replicate runs (errors in replicates).

One offspring had however only one successful MHCII‐MiSeq amplicon. Here, we called putative alleles if present in one or both parents in the same run.

### 2.3 Comparing genotypes from modified Sommer pipeline and AmpliSAS

**Figure 4:**
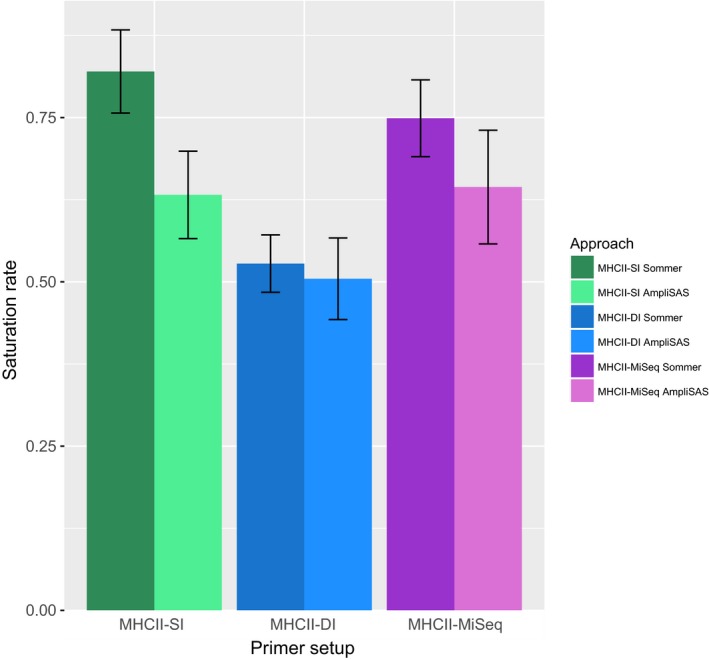
The saturation rate for each approach for genotyping MHC class II exon 2 in eight bluethroats, calculated as the average proportion of the individual “combined genotypes” each approach was able to genotype. The “combined genotype” was established for each individual by combining all alleles that were called in the individual using at least one approach

As the sequences were trimmed in the modified pipeline from Sommer et al. (2013) in order to match published sequences, the sequences from AmpliSAS were aligned and trimmed correspondingly in MEGA7, and collapsed using fastx toolkit. Identical alleles obtained in different pipelines were given identical names using standard UNIX commands. The genotypes were then compared, both among the different primer setups and between the allele‐calling pipelines, by counting shared alleles across methods within individuals. A saturation rate plot was established, to visualize the average proportion of all alleles genotyped for an individual each approach was able to genotype (Figure 4). The figure was made in R, version 3.2.5 (R Core Team 2016), with the package ggplot2 (Wickham, 2009).

Within each approach, the family data were evaluated in order to reveal lack of Mendelian inheritance, that is, variants scored as alleles in offspring but not in any of its parent. Errors in pedigree were calculated as the percentage of alleles genotyped in offspring individuals but not found in any of their parents. Only offspring having no failed replicates were used for this purpose.

## 3 RESULTS

### 3.1 MHCIe3

After barcode splitting in both allele‐calling pipelines, all MHCI‐SI amplicons and 12 of 16 MHCI‐DI amplicons had >5,000 reads per amplicon. The remaining four MHCI‐DI amplicons had >2,900 reads per amplicon.

Using the modified pipeline from Sommer et al. (2013), 18 unique alleles were identified in all eight individuals combined, with complete concordance for every individual between the MHCI‐SI and the MHCI‐DI primer setup. There were between five and eight alleles per individual across all samples (Appendix S7), corresponding to at least four MHCI‐loci. The 18 alleles translated into 14 unique amino acid sequences (Table 1, Appendix S8).

**Table 1:** Results from genotyping eight bluethroats at MHC class I exon 3 (MHCI), using two primer strategies (single index [SI] and dual index [DI]) on Ion Torrent

	MHCI‐SI	MHCI‐DI
**Modified pipeline from Sommer et al.** (2013)		
Reads per amplicon after jMHC (including only variants with ≥3 reads within any amplicon)	8,744–48,840	2,953–18,154
Total number of alleles	18	18
Alleles per individual	6.6 (5–8)	6.6 (5–8)
Unique amino acid sequences	14	14
Errors in pedigree	0	0
**AmpliSAS**		
Reads per amplicon	>5,000	3,002–>5,000
Reads per amplicon assigned to alleles	2,797–3,534	2,028–4,815
Total number of alleles	16	16
Alleles per individual	5.4 (4–7)	5.0 (4–7)
Alleles per amplicon	5.6 (4–7)	5.5 (4–8)
Unique amino acid sequences	13	13
Errors in replicates	3.4%	9.1%
Errors in pedigree	0	0

The datasets were genotyped through two pipelines; one using a modification of the pipeline published by Sommer et al. (2013) and one using the online tool AmpliSAS (Sebastian et al., 2016).

Two alleles were recognized by the modified Sommer pipeline only. Of these, one allele was not found in both replicates of any individual by AmpliSAS, while the other allele was marked as “suspicious sequence” by AmpliCHECK and not outputted in the final result from AmpliSAS. All alleles identified with AmpliSAS were also called using the modified Sommer pipeline.

Genotyping with AmpliSAS thus identified 16 alleles in total (Table 1), with the same unique variants genotyped using MHCI‐SI and MHCI‐DI. Three alleles found in an individual by MHCI‐SI were however not called as alleles in the same individual by MHCI‐DI, as they were found in only one of the two MHCI‐DI amplicons by AmpliSAS. Apart from this discrepancy, there was no disagreement between the results from the two primer setups (see Appendix S9).

There was no deviation from Mendelian inheritance of offspring alleles in either of the two allele‐calling pipelines.

### 3.2 MHCIIβe2

All Ion Torrent MHCII amplicons (MHCII‐SI and MHCII‐DI) and 12 of the 16 MHCII‐MiSeq amplicons had >5,000 reads after barcode splitting in both pipelines. Excluding one failed sample (one replicate MHCII‐MiSeq run of individual 69390), the remaining MHCII‐MiSeq amplicons had each assigned >2,400 reads at this stage.

#### 3.2.1 Modified pipeline from Sommer et al. (2013)

Analyzing the data using the modified pipeline from Sommer et al. (2013), 117 unique alleles were identified across all sequencing strategies. The 117 alleles translated into 105 unique amino acid sequences. Less than 5% of the alleles found in the offspring were not found in any of their parents (Table 2).

**Table 2:** Results from genotyping eight bluethroats at MHC class II exon 2 (MHCII), using two primer strategies (single index [SI] and dual index [DI]) on Ion Torrent as well as being sequenced on Illumina MiSeq

	MHCII‐SI	MHCII‐DI	MHCII‐MiSeq
**Modified pipeline from Sommer et al.** (2013)			
Reads per amplicon after jMHC (including only variants with ≥3 reads within any amplicon)	6,495–19,886	14,777–68,524	2,436–10,553
Total number of alleles	96	74	105
Alleles per individual	35.6 (29–47)	23.0 (17–31)	32.6 (23–41)
Unique amino acid sequences	84	65	93
Errors in pedigree	2.1%	4.3%	3.4%
**AmpliSAS**			
Reads per amplicon	>5,000	>5,000	2,877–>5,000
Reads per amplicon assigned to alleles	3,251–4,445	3,754–4,681	2,611–4,721
Total number of alleles	75	74	94
Alleles per individual	27.8 (20–39)	22.0 (16–30)	27.8 (21–34)
Alleles per amplicon	31.6 (22–42)	23.5 (16–31)	28.5 (21–35)
Unique amino acid sequences	68	64	82
Errors in replicates	12.1%	6.4%	8.4%
Errors in pedigree	6.4%	10.9%	12.5%

The datasets were genotyped through two pipelines; one using a modification of the pipeline published by Sommer et al. (2013) and one using the online tool AmpliSAS (Sebastian et al., 2016).

Fewer alleles were called in every individual using the MHCII‐DI sequencing approach, than in the two other approaches (averagely 35.6, 23.0, and 32.6 alleles were called per individual for the MHCII‐SI, MHCII‐DI, and MHCII‐MiSeq data, respectively). The number of alleles per individual called within the separate sequencing approaches ranged from 17 to 47. When combining all three sequencing approaches, a maximum of 25 alleles were genotyped per individual by all three approaches, while a maximum of 53 alleles were genotyped per individual in at least one approach.

#### 3.2.2 AmpliSAS pipeline

Across all eight samples and all three sequencing approaches, 114 unique alleles were called using AmpliSAS, which translated into 102 unique amino acid sequences. For this allele‐calling pipeline, 7–10 (6.4%–12.5%) pedigree errors were found in each of the primer approaches.

On average, the MHCII‐DI approach yielded 22.0 alleles per individual, while 27.8 alleles were scored per individual in both the MHCII‐SI and the MHCII‐MiSeq approaches. As with the modified Sommer pipeline, AmpliSAS also called fewer alleles using MHCII‐DI than MHCII‐SI for most individuals (in all individuals except two; on average 5.75 more alleles were scored per individual with MHCII‐SI than with MHCII‐DI).

Within the separate sequencing approaches, 16–39 alleles were found per individual (Table 2). Genotyping rendered a maximum of 20 alleles per individual found in all three sequencing approaches, and a maximum of 53 alleles per individual were found in at least one approach when combining the results.

#### 3.2.3 Comparing the approaches

We established a “combined genotype” for each individual consisting of all unique MHCIIβe2 alleles that were called in at least one of the approaches for that respective individual. The number of alleles per individual in the “combined genotype” ranged from 35 to 56, with an average of 43.5. Averaged over all individuals, the MHCII‐SI primer setup followed by allele calling with the modified Sommer pipeline was able to retrieve the highest percentage of the “combined genotype” (i.e., saturation rate), as compared to the other approaches (Figure 4). The dual‐indexed approaches had the lowest saturation rate, while within every primer setup, the modified Sommer pipeline had higher saturation rate than when allele calling using AmpliSAS. The maximum number of unique alleles in a combined genotype (i.e., 56 alleles) implies a minimum of 28 MHCIIβe2 loci in the bluethroat (see Appendix S10).

All unique alleles across all individuals found with AmpliSAS were also found using the modified Sommer pipeline, except for one allele which was also the only allele lacking a cysteine residue in position 75 when translated—which suggests that it is a nonfunctional allele (see Appendix S11). Within each individual, on average 80.1% (MHCII‐SI), 94.4% (MHCII‐DI), and 91.9% (MHCII‐MiSeq) of the alleles were called by both the modified Sommer pipeline and AmpliSAS. For the MiSeq data, all alleles genotyped for each individual by AmpliSAS were also called by the modified Sommer pipeline. The latter pipeline genotyped on average 4.88 more MiSeq alleles per individual than AmpliSAS.

For both allele‐calling pipelines, fewest unique alleles were genotyped across all samples using the MHCII‐DI data, while the highest number of unique alleles was found in the MiSeq dataset. Also in terms of alleles per individual, the MHCII‐DI run rendered fewest alleles for both pipelines (Table 2 and Figure 4).

## 4 DISCUSSION

In this study, we aimed to establish a robust genotyping method for MHC class I exon 3 (MHCIe3) and MHC class II exon 2 (MHCIIβe2) in bluethroats. Simultaneously, we intended to highlight possible differences in MHC genotyping resulting from different sequencing platforms, primer design, and bioinformatic allele‐calling pipelines. For the hypervariable MHCIIβe2, both the number and the identity of alleles varied among the abovementioned approaches. More consistent genotypes were obtained when analyzing MHCIe3, in which mainly bioinformatic pipeline but not primer structure influenced the results. Our use of family data and replicates was advantageous in order to validate alleles. We thus recommend including such data when analyzing highly polymorphic markers.

### 4.1 Sources of variation among strategies: platforms

One of the main challenges when genotyping variable multilocus systems like MHC is to be able to separate real alleles from artifacts (Babik et al., 2009; Lighten et al., 2014). As specific sequencing platforms have typical error profiles (Duke et al., 2015; Loman et al., 2012; Quail et al., 2012)—for example, homopolymer errors are more prominent for Ion Torrent than Illumina—different platforms could render different genotypes (Sebastian et al., 2016). Variant filtering in subsequent allele‐calling pipelines is however supposed to eradicate sequencing errors. Yet, this process might not work perfectly and could potentially result in high‐depth, in‐frame artifactual variants incorrectly being called as alleles. Nevertheless, the low number of alleles not following Mendelian inheritance makes this unlikely to be the only explanation for the observed differences in genotypes. Another reason for the discrepancy could be platform‐specific adapter motifs in the primers (mechanisms for unequal amplification discussed below).

### 4.2 Sources of variation among strategies: primers

The differences between primer approaches are better elucidated when comparing Ion Torrent runs, which are not confounded by differences in sequencing platforms. It is important to note that the MHCIe3 runs with different primer structure (MHCI‐SI and MHCI‐DI) yielded identical results within each allele‐calling pipeline (except one individual genotyped with AmpliSAS; discussed later), while this was not the case for MHCIIβe2 (MHCII‐SI and MHCII‐DI).

The underlying mechanisms that possibly explain the observed MHCIIβe2 genotype discrepancies may involve differential amplification of alleles (e.g., Sommer et al., 2013). A “tail” of spacer/barcode/adapter in the primer sequence would preferentially amplify variants that have complementary bases to this tail outside the gene target. Such a tail is applied on both forward and reverse primers in the DI approach, while in contrast, the SI primers have only the barcode attached, and only to the forward primers (Ion Torrent adapters were ligated onto the amplicons after MHC amplification). As a result, we would expect a stronger effect and fewer alleles to be amplified using the DI approach. Indeed, we observed fewer alleles called for every individual by both bioinformatic pipelines using MHCII‐DI than MHCII‐SI, when disregarding alleles that exhibited Mendelian errors.

Amplified PCR fragments will have complementary sequences to the whole primer (including the tail), in contrast to the template, in which only the gene target is complementary to the primer sequence. The annealing affinity of the primers in the PCR will thus be higher to amplified fragments compared to template sequences. Because the DI primer setup consists of a longer tail on both forward and reverse primers, this “affinity effect” may create more bias and potential allelic dropouts in the MHCII‐DI amplifications than in the MHCII‐SI amplifications.

Lastly, the probability of secondary structure formation in the DI primer sequence is higher due to the longer primer tail. The combination of these effects might explain the lower number of observed alleles for the MHCII‐DI approach compared to the MHCII‐SI approach.

Our results demonstrate that the use of phusion primers can create allelic dropout in PCR amplifications of multilocus targets. Researchers should be aware of this potential pitfall and address this issue when sequencing polymorphic multilocus regions such as the MHC.

### 4.3 Sources of variation among strategies: allele‐calling pipelines

Within each MHCIIβe2 primer setup, we found distinct genotypes depending on the allele‐calling pipelines used. First, one possible cause to the disparity between the modified Sommer pipeline and AmpliSAS is the clustering in the AmpliSAS algorithm. While all variants passing the filters are called as alleles in the modified Sommer pipeline, AmpliSAS cluster similar variants based on platform‐specific error rates and relative frequencies of variants clustered together. Hence, the fewer alleles scored using AmpliSAS could be caused by erroneous clustering of low‐frequency, true alleles to other true alleles. The discrepancy could also arise from high‐frequency artifacts incorrectly being called as alleles in the modified Sommer pipeline. The latter is unlikely to be a general explanation because all except two of the 39 MHCIIβe2 alleles called using the modified Sommer pipeline and not AmpliSAS showed Mendelian inheritance. However, repeatable errors could be a cause of this pattern. Checking the “=1 bp”‐variants in offspring revealed that many were instances where the “=1 bp” variant was found within one parent which lacked the “source” variant, while the “source” variant was found within the other parent which lacked the “=1 bp” variant (data not shown). Repeatable errors are thus likely not a major problem in the modified Sommer pipeline, although we cannot dismiss it completely. Also, within each MHCIIβe2 primer setup, not all of the alleles called only when using the modified Sommer pipeline had high sequence similarities to other alleles (see Appendix S12). Clustering of similar alleles may thus not account for all the instances in which AmpliSAS genotyped fewer alleles.

Second, the use of replicates could be an additional explanation for the higher number of alleles called when using the modified Sommer pipeline. In the AmpliSAS pipeline, we scored a variant as an allele in an individual if the AmpliSAS program genotyped it as an allele for both replicates. Variants found in only one of the replicates were however treated more carefully in the modified Sommer pipeline. Here, such variants were called as alleles if the variant in question was more than one base pair different from a more frequent variant within the same amplicon and concurrently found within other amplicons of the same family group. The underlying rationale is that these “>1 bp variants” are less likely to be sequencing errors, but if they are, it is unlikely that the same artifact is found within multiple amplicons from the same family group. Variants that are only one base pair different from a more frequent variant are managed more strictly and are required to be present in both replicates of an individual in order to be called as allele.

The use of replicates can also affect the interpretation of chimeras, and hence the number of alleles called. Whereas a variant that is scored as a putative allele in one amplicon but marked as a chimera in the replicate will be called as an allele in the modified Sommer pipeline, this variant will not be genotyped by AmpliSAS. This is because chimeras are removed from the output from AmpliSAS, and as the variant is then lacking from one of the two replicates, it will not be called as an allele.

While we reduced the number of PCR cycles to 25 in order to minimize PCR artifacts, other actions could be taken, for instance a prolonged elongation step or the introduction of a reconditioning step (Lenz & Becker, 2008). However, as we are comparing approaches that all apply the same PCR‐protocol (see Appendix S5), this is not further evaluated here.

Third, there could be an effect of difference in coverage levels required for the two allele‐calling pipelines. AmpliSAS is based on a subsampling of 5,000 reads from each amplicon as default, while the modified Sommer pipeline takes all reads within an amplicon into account. Biedrzycka et al. (2017) achieved high repeatability and reliability when using 5,000 reads per amplicon for genotyping a sedge warbler MHCI‐dataset (with complexity similar to the MHCIIβe2 dataset in our study) by AmpliSAS, justifying the use of the default subsampling value. Implementing a minimum amplification efficiency of 0.2 (as in Biedrzycka et al. (2017)) and a maximum of 47 alleles per individual to the information in Figure S6 and Table S4 in Sommer et al. (2013), a minimum of 2,456 reads per amplicon are required to determine a complete genotype with at least three reads per allele (99.9% confidence level). As all MHCIIβe2 amplicons sequenced on the Ion Torrent initially had >5,000 reads each, while all except one (<500 reads) MHCII‐MiSeq and MHCI amplicons had >2,800 reads, we chose to only exclude the one failed MiSeq amplicon. While the remaining amplicons thus would have sufficient coverage for genotyping using the modified Sommer pipeline, we also chose to keep these amplicons for AmpliSAS, because of the additional strength we get from including family data and replicates of each individual. The missing of a MHCIIβe2 allele in AmpliSAS could thus be due to low coverage, as three of the eight individuals had one MHCII‐MiSeq amplicon replicate with coverage <5,000 (2,877–4,399) reads. Indeed, eight of the 11 MiSeq alleles found by the modified Sommer pipeline and not by AmpliSAS could be explained in this manner, where the alleles missing when genotyping in AmpliSAS are found in high‐coverage amplicons but not in their lower‐coverage replicates (data not shown). In other words, this implies that higher sequencing depth is required for the AmpliSAS pipeline compared to the modified Sommer pipeline in order to obtain the same accuracy when genotyping highly polymorphic loci. Bluethroat MHCIe3, which has relatively few loci (i.e., four; O'Connor et al., 2016), is likely not affected by coverage differences to the same extent as the polymorphic bluethroat MHCIIβe2. This is in line with Razali, O'Connor, Drews, Burke, and Westerdahl (2017), who found that MiSeq and 454 sequencing provided equal results despite differences in read depths when genotyping amplicons with low diversity, while the results were less consistent in amplicons with higher diversity.

### 4.4 Comparing the approaches

Genotyping MHCIe3 yielded mainly consistent results across all approaches. Still, the modified Sommer pipeline revealed two additional unique alleles compared to the results from AmpliSAS. These alleles showed neither any deviation from Mendelian inheritance nor any signs of nonfunctionality, and we assume they are false negatives in the AmpliSAS pipeline. Further, within each allele‐calling pipeline, only one individual exhibited differences in MHCI‐SI and MHCI‐DI genotypes (genotyped using AmpliSAS). The three “missing” alleles in the MHCI‐DI genotype were however called in one of the MHCI‐DI replicates and found in the unfiltered AmpliSAS results for both replicates. The lack of these MHCI‐DI alleles could thus be caused by erroneous filtering in AmpliSAS.

For the hypervariable MHCIIβe2, the results were less consistent. Across all primer approaches, four unique alleles were found only when using the modified Sommer pipeline, while one unique allele was found only using AmpliSAS. It is worth mentioning that this latter allele was the only allele lacking the important cysteine residue in position 75 and hence is likely a pseudogene or an artifact. As cysteine residues in position 10 and 75 are included in the filtering steps of the modified Sommer pipeline, this allele would not be retained in the outputted genotype.

Within each primer setup, more MHCIIβe2 alleles were scored using the modified Sommer pipeline than AmpliSAS. This is evident also from the saturation rate plot (Figure 4; see also Appendix S13), where a higher percentage of the combined genotypes (i.e., alleles called within an individual using at least one approach) were called using the modified Sommer pipeline than using AmpliSAS. Most of these alleles are expected to be true positives, as almost all alleles called in the offspring also were genotyped for one or both of their parents (Table 2). Furthermore, there were fewer errors in pedigree for genotyping with the modified Sommer pipeline than with AmpliSAS. These numbers are however not directly comparable between the allele‐calling pipelines, because of the use of family information to facilitate genotyping in the modified Sommer pipeline. The “errors in pedigree” (Table 2) will thus be biased towards the modified Sommer pipeline as compared to AmpliSAS. Yet, the lower number of errors in pedigree and the higher saturation rate still suggest that the modified Sommer pipeline could render more consistent and comprehensive results than AmpliSAS. The more automated allele calling through AmpliSAS is however both faster and less prone to human mistakes, which need to be taken into account when deciding upon which approach to use. Based on our results, we still recommend the use of the modified Sommer pipeline on highly polymorphic systems, especially when family data are available and the sequencing is not ultradeep (>5,000 reads in every amplicon).

Correspondingly, MHCII‐SI (genotyped using the modified Sommer pipeline) had higher saturation rate and lower percentage of errors in pedigree compared to the other approaches and could be the preferred approach. However, single indexing requires substantially more barcodes when multiplexing a large number of individuals, and sequencing using dual‐indexed primers on Illumina MiSeq as in this study (Figure 1) could thus be a cost‐efficient option.

The difficulty of correctly genotyping highly polymorphic loci like bluethroat MHCIIβe2 also raises the issue of balancing false negatives against false positives. An approach that calls more alleles will likely also score some artifacts as alleles, while a “stricter” approach will be more prone to fail to genotype true alleles. The relative importance of this will likely be dependent on the research question (e.g., whether the study is on diversity, or associations between pathogens and MHC alleles). Although false positives such as repeatable errors cannot be completely ruled out, we believe that consistency in family data is an overall strength for the approach used. Hence, the opportunity for validation of alleles provided by family data is of great value, and using an approach that minimizes the errors in inheritance pattern could guide the choice of method.

### 4.5 Polymorphism levels in MHCIe3 and MHCIIβe2 in bluethroats

For MHC class I, we detected maximum eight alleles per individual, implying minimum four MHCIe3 loci in bluethroats. This is in accordance with the results from O'Connor et al. (2016), who also reported four loci in the species, using a dual index approach and two different primer combinations. The same study revealed considerable diversity in the number of MHCIe3 loci across passerines, with bluethroats (four loci) and willow warblers (19 loci) at the extreme ends. This strengthens our findings of relative low complexity at MHCI in bluethroats, and that the genotyping at these loci likely is robust against variation in primer design and allele‐calling approaches in the species.

The complexity recognized at MHCIIβe2 in this study is in stark contrast to the results from MHCIe3. Our study supports the findings of both Anmarkrud et al. (2010) and Gohli et al. (2013), where high levels of MHCIIβe2 polymorphism were detected in the bluethroats through cloning and Sanger sequencing. Although likely underestimated due to technical limitations, Anmarkrud et al. (2010) identified a minimum of 11 functional MHCIIβe2 loci. In this study, when combining all strategies, up to 56 alleles and thus a minimum of 28 MHCIIβe2 loci were described for one individual, testifying to the incredible diversity at this marker.

## 5 CONCLUSION

Our results reveal that different genotyping strategies yield similar genotypes in bluethroat MHCIe3, a system with relatively low polymorphism. In contrast, caution needs to be exercised when sequencing highly complex markers such as the bluethroat MHCIIβe2. For bluethroat MHCIIβe2, our results demonstrate that genotyped alleles will be biased according to both primer design and allele‐calling pipeline. Consequently, comparisons of results across approaches and studies are error prone in this polymorphic marker. However, the use of family data and replicates lends support to results found within each strategy and prove to be especially valuable for validation of alleles in the complex MHCIIβe2. As such, the methodology described herein could be useful for exploration of ecological and evolutionary relevant hypotheses relative to MHC variation, even though it does not necessarily describe the true repertoire of alleles within each individual.

## CONFLICT OF INTERESTS

The authors declare no conflict of interests.

## AUTHOR CONTRIBUTIONS

SLR, JAA, AJ, JTL designed the study. JAA performed the laboratory experiments. SLR performed the bioinformatics analyses and drafted the manuscript. All authors reviewed the manuscript and approved the final version.

## DATA ACCESSIBILITY

MHC alleles uncovered in this study have been imported to GenBank (accession nos. MF769960–MF769977 (MHCIe3) and accession nos. MF769842–MF769959 (MHCIIβe2)). All sequence data used in this study are uploaded to the NCBI Sequence Read Archive under BioProject ID: PRNA400123. Voucher specimen accession nos. and barcodes: See Appendix S1. Each voucher accession no. is searchable via the online Collection Explorer: http://nhmo-birds.collectionexplorer.org/accession.aspx.
