# Supplementary material for: Genotyping strategy matters when analyzing hypervariable major histocompatibility complex‐Experience from a passerine bird
Source: Ecol Evol. 2018 Jan 7;8(3):1680–92. doi: 10.1002/ece3.3757 (PMC5792522; doi:10.1002/ece3.3757)
Supplement: Supplementary file 2 [file ECE3-8-1680-s002.docx]

| Term used in this paper | Explanation |
| --- | --- |
| Read | A single, not necessarily unique, DNA-sequence derived from sequencing |
| Amplicon | DNA fragments amplified from a single marker in one PCR, from a single individual with a unique sequence tag |
| Variant | Cluster of identical reads |
| Allele | Variants that are passing all filters, and are believed to be a protein coding MHC sequence |
| Artefact | Variants that are not called as alleles in an individual, due to sequencing errors, shift in reading frame *et cetera* |
| Replicate | Replicates are amplicons derived from the same DNA extract, but that are amplified in independent PCRs |
