# Supplementary figures and images for: Genotyping strategy matters when analyzing hypervariable major histocompatibility complex‐Experience from a passerine bird
Source: Ecol Evol. 2018 Jan 7;8(3):1680–92. doi: 10.1002/ece3.3757 (PMC5792522; doi:10.1002/ece3.3757)

# PRIMER BINDING SITES

## MHCI

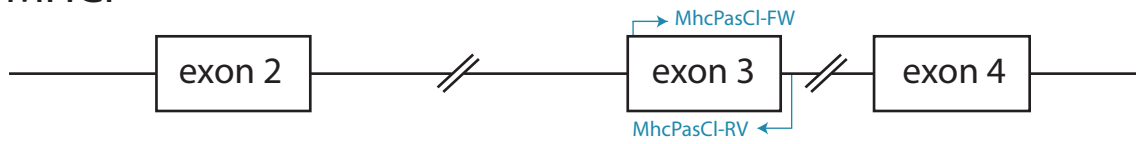

## MHCII

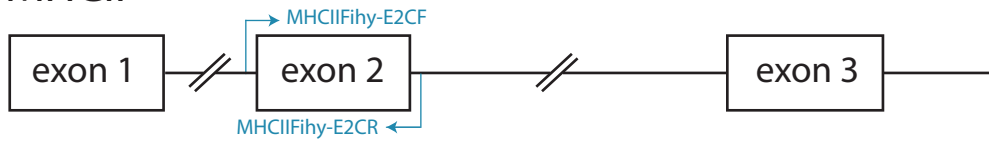

Modified from Alcaide *et al.* (2013; MHC I) and Canal *et al.* (2010; MHC II)

Supplement: Supplementary file 4 [file ECE3-8-1680-s004.pdf]

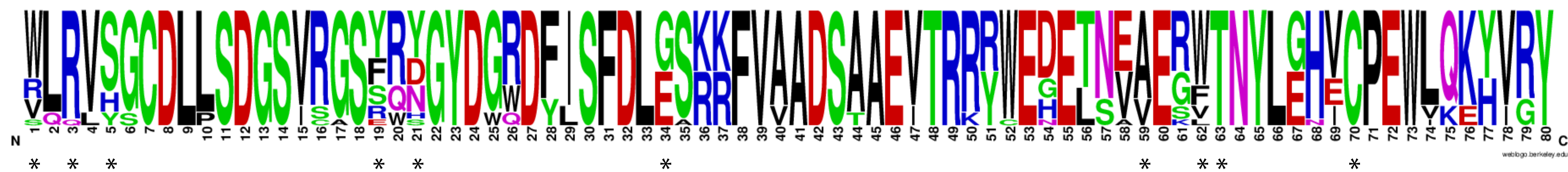

Supplement: Supplementary file 8 [file ECE3-8-1680-s008.pdf]

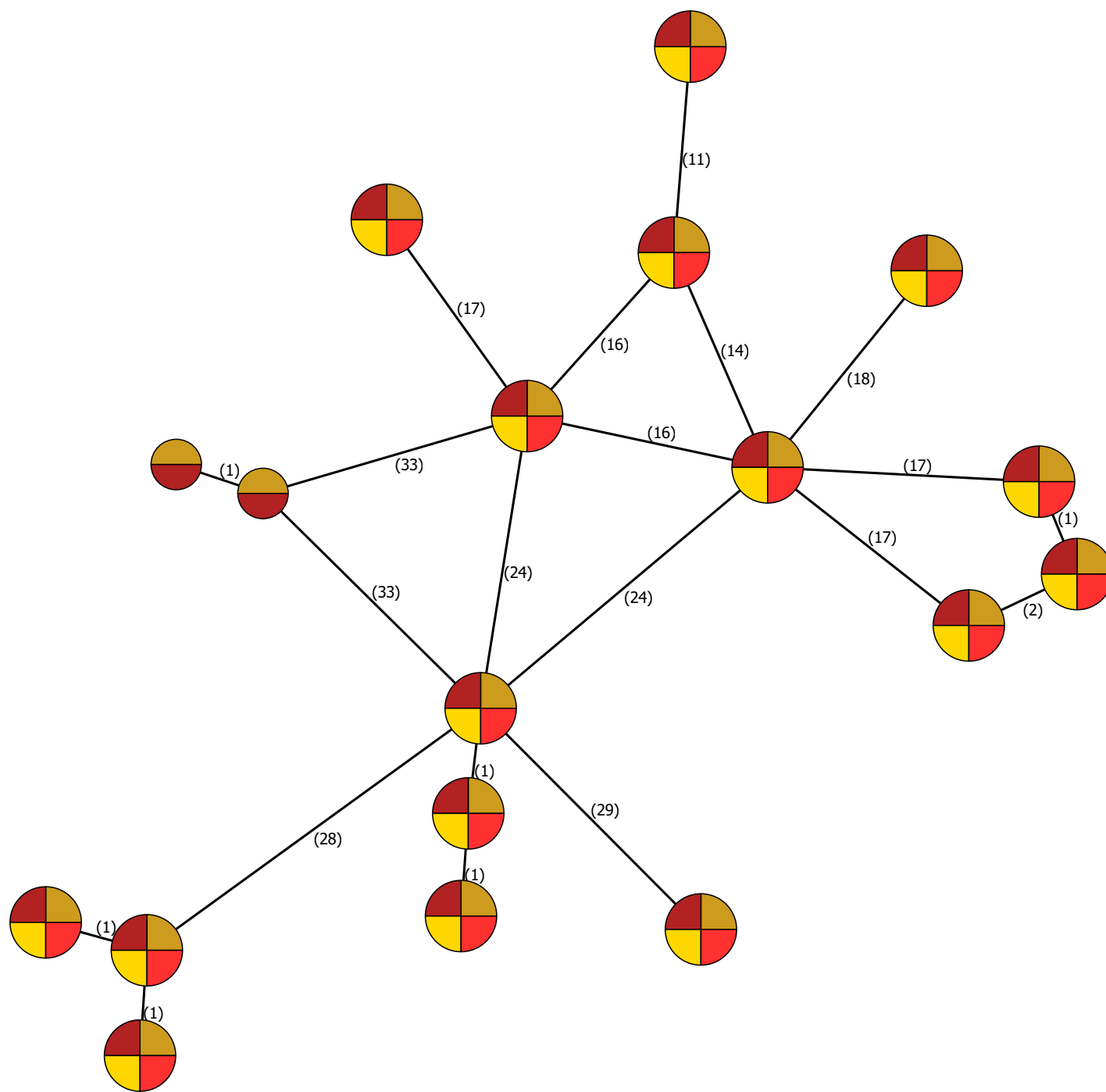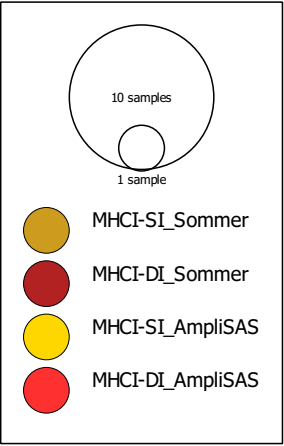

Supplement: Supplementary file 9 [file ECE3-8-1680-s009.pdf]

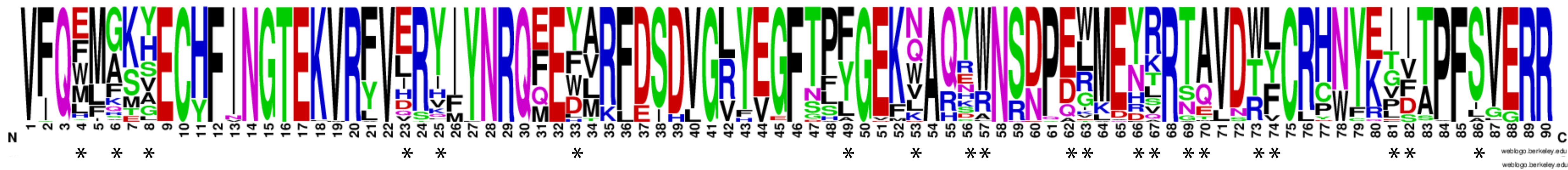

Supplement: Supplementary file 11 [file ECE3-8-1680-s011.pdf]

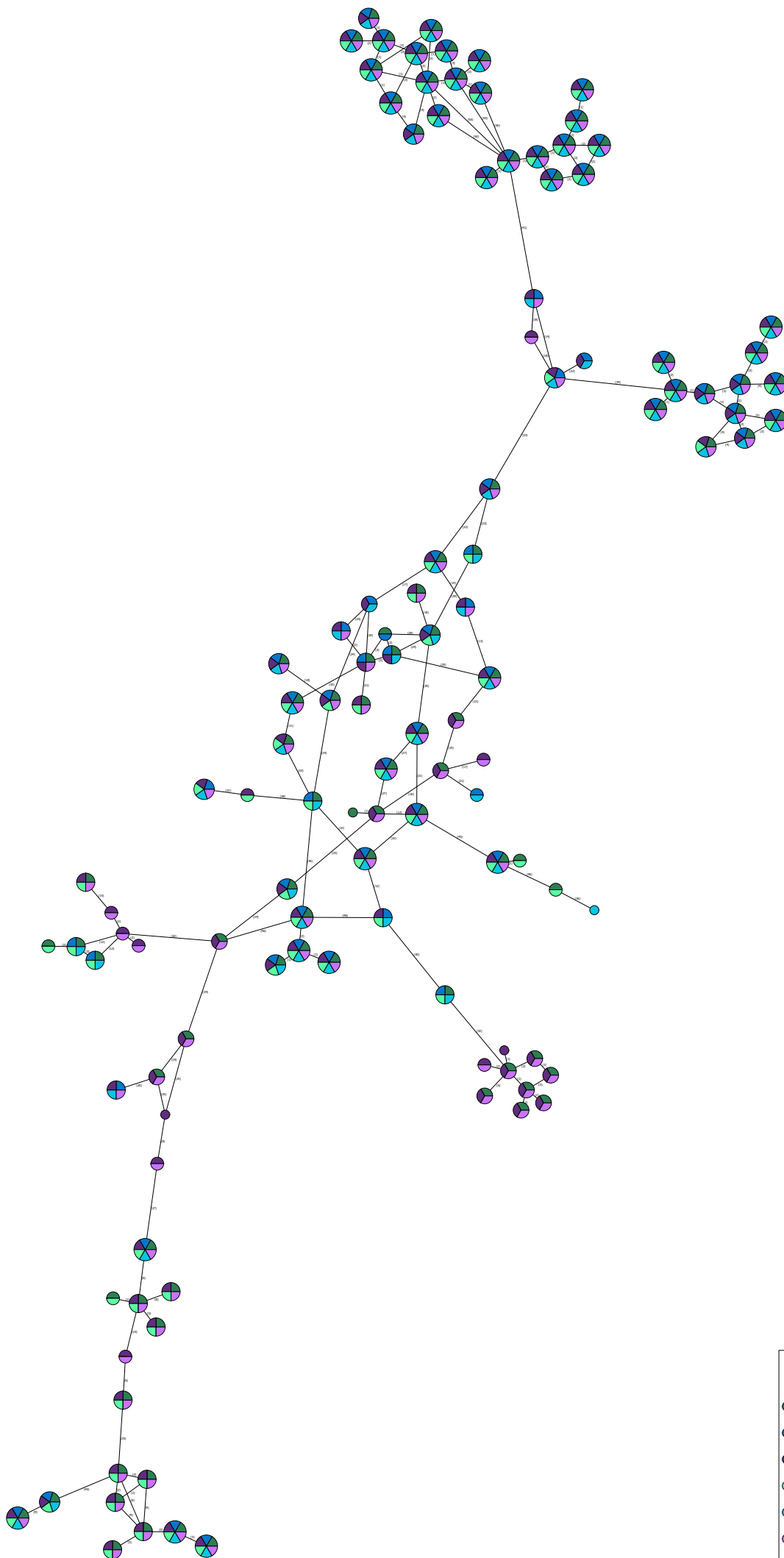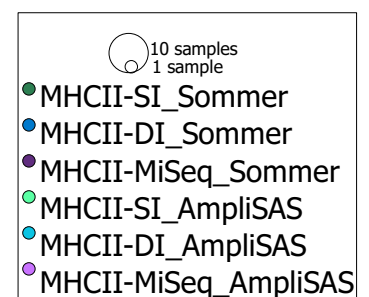

Supplement: Supplementary file 12 [file ECE3-8-1680-s012.pdf]

## AmpliSAS

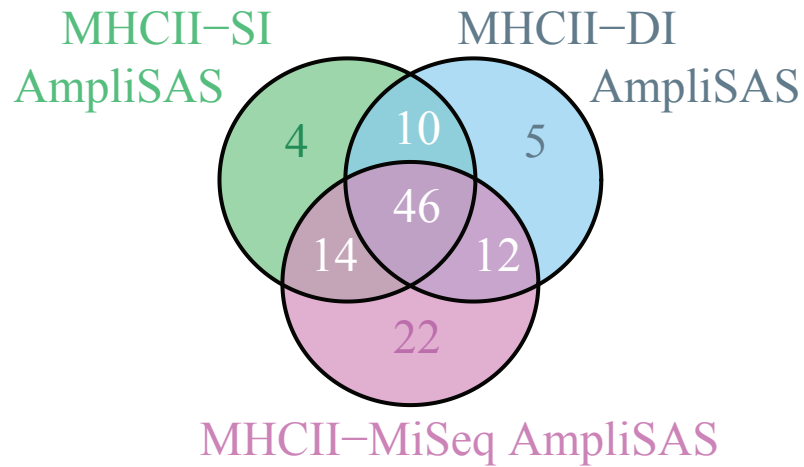

## Modified Sommer pipeline

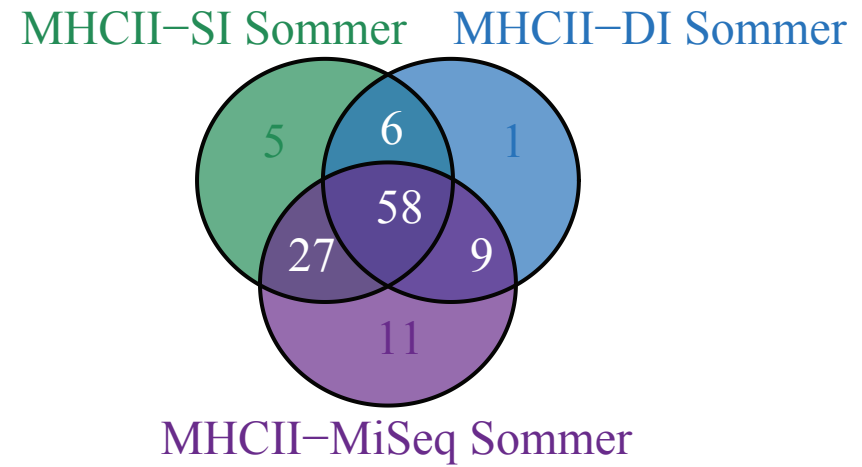

## MHCII-MiSeq Alleles

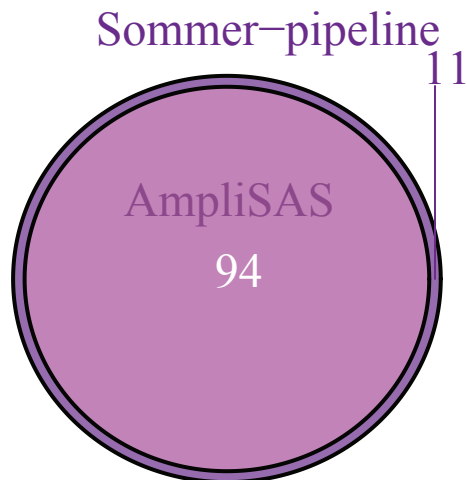

## MHCII IonTorrent Alleles

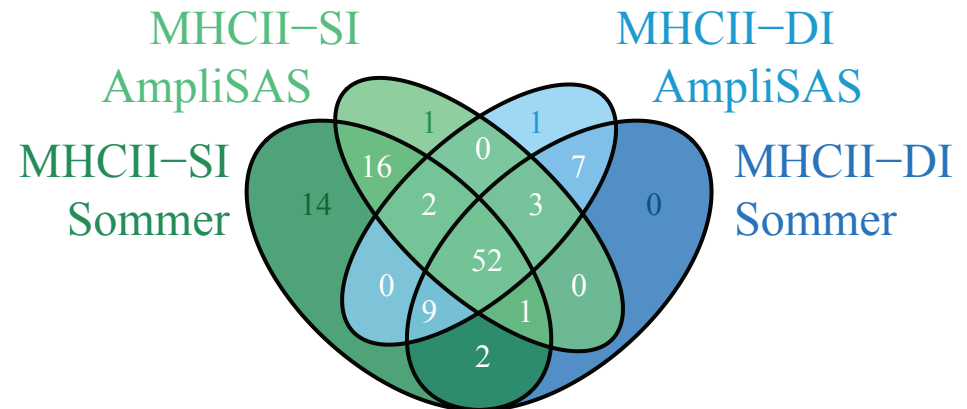

Supplement: Supplementary file 13 [file ECE3-8-1680-s013.pdf]
