# Supplementary material for: Genotyping strategy matters when analyzing hypervariable major histocompatibility complex‐Experience from a passerine bird
Source: Ecol Evol. 2018 Jan 7;8(3):1680–92. doi: 10.1002/ece3.3757 (PMC5792522; doi:10.1002/ece3.3757)
Supplement: Supplementary file 5 [file ECE3-8-1680-s005.docx]

# Sequencing of MHCI exon 3 and MHCII exon 2 in bluethroats

### MHC class I exon 3 sequencing using IonTorrent

Single-indexed amplicons (MHCI-SI)

MHC class I exon 3 amplicons were generated using the MhcPasCI-FW and MhcPasCI-RV primer pair (Alcaide et al. 2013). The forward primers were combined with IonXpress barcodes and the GAT primer adapter motif (see Appendix S3). The amplicons were amplified with the following conditions: 1X reaction buffer, 0.8 mM dNTP, 0.5 µM of each primer and the Q5 polymerase (New England Biolabs) following the manufacturer’s recommendations. Additionally, we included 1X Q5 High GC Enhancer supplied with the polymerase for this PCR reaction. The following thermal profile was applied: initial denaturation at 98 °C for 30 seconds, 25 cycles of denaturation 98 °C for 10 seconds, annealing at 59 °C for 20 seconds and elongation at 72 °C for 15 seconds, and a final elongation step at 72 °C for 2 minutes. Amplicons were inspected on agarose gels and concentrations were estimated on a Fragment Analyzer (Advanced Analytical) using the High Sensitivity Genomic DNA Analysis kit (Advanced Analytical). Equimolar amounts of each amplicon were pooled and the final library was prepared using the NEBNext® Fast DNA Library Prep Set for Ion Torrent (New England Biolabs) following the manufacturers recommendations. The amplicons were sequenced on a 316v2 chip prepared using Ion Chef (ThermoFischer) on an Ion Personal Genomic Machine (PGM) (ThermoFischer) together with the dual-indexed IonTorrent libraries and 12 amplicons not included in this project (60 amplicons in total).

Dual-indexed amplicons (MHCI-DI)

The dual-indexed amplicons were generated with the same gene target as for the single-indexed amplicons. Yet, the amplification primers were designed to include the following motifs: (i) IonTorrent A adapters on the forward primers and IonTorrent trP1 adapters on the reverse primers, (ii) A unique combination of IonXpress barcode on forward primers and reverse complement IonXpress barcode on reverse barcodes, (iii) barcode adapter motif GAT, (iv) a “spacer motif” of seven nucleotides, as described in Fadrosh et al. (2014). The motivation for including this motif was to mimic amplicons prepared for Illumina sequencing (see below) and to be able to distinguish single-indexed and dual-indexed MHC class I exon 3 amplicons, (v) Gene target primers.

The amplicons were obtained using the same PCR conditions and thermal profile as for the single-indexed amplicons. Amplicon concentrations was estimated on a Fragment Analyzer (Advanced Analytical) using the High Sensitivity Genomic DNA Analysis kit. Equimolar amounts of amplicons were pooled and purified using Agencourt AMPure XP beads (Beckman Coulter, Inc.) and sequenced directly on a 316v2 chip, prepared using Ion Chef (ThermoFischer) (together with the MHCI-SI and MHCII-DI libraries as described above), on an Ion PGM system (ThermoFischer).

### MHC class II exon 2 amplicon sequencing using IonTorrent

Single-indexed amplicons (MHCII-SI)

For the single-indexed amplicons, we used the MHCIIFihy-E2CF and MHCIIFihy-E2CR primers described by Canal et al. (2010) as gene target. IonXpress barcodes were included together with the barcode adapter GAT on the forward primer sequences. PCR conditions and thermal profile were the same as for MHC class I exon 3, with the exception of annealing at 72 °C and exclusion of the Q5 High GC Enhancer. All amplicons were inspected on agarose gels. To prepare the sequencing library, we followed the same procedure as described for the MHC class I exon 3 single-indexed library. The library was sequenced along with four additional amplicons on a 316v2 chip prepared using the Ion OneTouch 2 system (ThermoFischer) using Ion PGM system (ThermoFischer).

Dual-indexed amplicons (MHCII-DI)

Dual-indexed primer strategy was the same as for the dual-indexed MHC class I exon 3 IonTorrent library, except for the use of MHCIIFihy-E2CF/MHCIIFIhy-E2CR (Canal et al. 2010) primer pair as gene target (see Appendix S3). PCR conditions and thermal profile were the same as for the MHC class II exon 2 single-indexed sequencing strategy. The final library was sequenced together with the MHC class I exon 3 amplicons as described above.

### MHC class II exon 2 amplicon sequencing using Illumina MiSeq *(MHCII-MiSeq)*

The 16 amplicons for Illumina MiSeq sequencing was generated using a dual-indexed sequencing strategy, together with 560 other amplicons (manuscript in prep.) The PCR amplification primer setup followed Fadrosh et al. (2014), which included in both forward and reverse direction: (i) Illumina Linker Sequence, (ii) a 12 nucleotide barcode, (iii) a “heterogeneity spacer” motif of 0-7 nucleotides for optimization of MiSeq amplicon sequencing, (iv) gene target primers. Unique combinations of 24 forward and 24 reverse barcodes allowed for multiplexing of 576 amplicons. Gene target motif was the same as for the single-indexed MHC class II exon 2 primers (see Appendix S3). PCR conditions and thermal profile was as described for the IonTorrent MHCII strategies. Amplicons were inspected on agarose gels, equimolar amounts of amplicons were pooled, purified and sequenced on an Illumina MiSeq® platform using v3 chemistry.

#### References:

Alcaide, M., Liu, M., & Edwards, S. V. (2013). Major histocompatibility complex class I evolution in songbirds: universal primers, rapid evolution and base compositional shifts in exon 3. *PeerJ,* 1, e86. doi: 10.7717/peerj.86

Canal, D., Alcaide, M., Anmarkrud, J. A., & Potti, J. (2010). Towards the simplification of MHC typing protocols: targeting classical MHC class II genes in a passerine, the pied flycatcher *Ficedula hypoleuca*. *BMC research notes,* 3, 236. doi: 10.1186/1756-0500-3-236

Fadrosh, D. W., Ma, B., Gajer, P., Sengamalay, N., Ott, S., Brotman, R. M., & Ravel, J. (2014). An improved dual-indexing approach for multiplexed 16S rRNA gene sequencing on the Illumina MiSeq platform. *Microbiome,* 2, 6. doi: 10.1186/2049-2618-2-6
