# Supplementary material for: Genotyping strategy matters when analyzing hypervariable major histocompatibility complex‐Experience from a passerine bird
Source: Ecol Evol. 2018 Jan 7;8(3):1680–92. doi: 10.1002/ece3.3757 (PMC5792522; doi:10.1002/ece3.3757)
Supplement: Supplementary file 6 [file ECE3-8-1680-s006.docx]

# Allele calling of MHCI exon 3 and MHCII exon 2 in bluethroats

## Modified pipeline from Sommer et al. (2013):

Initially, short reads (<150 base pair) were removed using the awk-command in UNIX (IonTorrent-data), and paired end reads were merged using FLASH v1.2.11 (Magoč and Salzberg (2011), MiSeq-data). Further raw read filtering was done using fastx toolkit v0.0.13/0.0.14 (http://hannonlab.cshl.edu/fastx_toolkit/), by removing reads where >5% of the bases had a Phred quality score below 20. The heterogenic spacer motifs in the MiSeq-data were removed by standard UNIX commands, in order to make the unique identifiers of equal lengths as demanded by jMHC (see below).

Of the 4.1 million raw reads obtained for IonTorrent run combining MHCI-SI, MHCI-DI and MHCII-DI (60 amplicons, see Appendix S5), 44.5 % passed the initial quality filtering. The MHCII-SI run rendered 3.5 million reads across all 20 amplicons sequences (see Appendix S5), of which 10.7% passed the initial quality filters. The MHCIIβe2 was also sequenced for the eight test-individuals at an Illumina MiSeq-platform together with a larger dataset consisting of duplicate samples of 280 additional individuals. FLASH combined 84.88% of the paired end raw reads and of these in total 9.9 million merged reads, 96.5% passed the quality filtering.

For all datasets, remaining reads were assigned to amplicons based on barcode, and clustered into variants using the software jMHC v1.6.1624 (Stuglik et al. 2011). Only variants with complete barcodes and three or more reads across the dataset were outputted. The variants were further evaluated in an Excel spreadsheet, and variants with less than three reads in any amplicon were discarded. Amplicons with less than 500 reads in total after this step were removed (applied to one MHCII-MiSeq amplicon).

One of the major modifications of the Sommer-pipeline was the implementation of a second frequency threshold above which most true alleles should occur, similar to Galan’s *T_2_* (Galan et al. 2010). A central assumptions when genotyping MHC using PCR-based high throughput sequencing methods is that most artifacts should have lower sequencing depths than real alleles (Babik et al. 2009; Lighten et al. 2014). As a first exploratory step of separating real alleles from artifacts, we thus set cut-off values based on the distribution of unique variants at different intra-amplicon threshold levels for inclusion (Figure 3, main text). By visually inspecting the plot, we tried to find the balance between including true alleles with low amplification efficiency and excluding true artefacts. The greatest change in the number of unique variants included was observed below 0.10 % - 0.20 %, suggesting the inclusion of low frequency artefacts around these cut-off values. Because the remaining variants would be filtered based on other criteria in subsequent steps of the pipeline, we applied a threshold value of 0.20 % in the analyses as an approach of classifying “possible alleles”. This value is close to the maximum per amplicon frequency of 0.18 % used by Biedrzycka et al. (2017) when examining a sedge warbler MHC dataset.

Further, every variant exceeding the threshold frequency in any amplicon was then aligned to published sequences from bluethroat (GenBank accession number KU169737-KU169747 (MHCIe3; O'Connor et al. (2016)) and HQ539575-HQ539614 (MHCIIβe2; Gohli et al. (2013)), using ClustalW (Thompson et al. 1994) in MEGA7 v7.0.14 (Kumar et al. 2016). All variants displaying shifts in reading frame or displaying stop codons when translated were removed, and bases outside the targeted 239 (MHCIe3) or 267 (MHCIIβe2) base pairs were deleted. Following O'Connor et al. (2016) and Gohli et al. (2013) respectively, we additionally removed variants not possessing Cys7 and Cys70 (MHCI) or Cys10 and Cys75 (MHCII), motivated by the conserved function of these residues. Identical variants were collapsed using the fastx_collapser command in fastx toolkit, and their depths were summed up.

The variants were sorted based on their intra-amplicon frequency. The most frequent variant in every amplicon was scored as “allele” for the amplicon in question. Chimera detection was conducted on the included variants of each amplicon in UCHIME (Edgar et al. 2011), using the uchime_denovo command in USEARCH v8.1.1861 or v 7.0.1090. For the non-chimeric variants, the number of base pair differences to the most similar, more frequent variant was calculated in MEGA7. As variants with only one base pair difference from a more frequent variant are more likely to be an artefactual sequence, these “=1bp”-variants were treated more strictly in the subsequent workflow. This is in line with the recommendations for species with “closely related ‘putative alleles’” (Sommer et al. 2013), which we are expecting for the bluethroat (MHCII; see Anmarkrud et al. (2010)).

In the next step, amplicon replicates and variant status (*i.e.* chimeric, “=1bp” and “>1bp” variants) were used to identify artifacts. First, a variant was considered an artifact in four scenarios: (1) a chimeric variant not present in amplicon replicate; (2) a chimeric variant detected as a chimera in amplicon replicate; (3) a “=1bp” variant not present in amplicon replicate; or (4) a “>1bp” variant not present in any other amplicon from individuals within the same family group. The artifact scoring was done for all amplicons before proceeding in the workflow. The variant was considered present if it had a frequency above the intra-amplicon threshold, and not only by its mere presence with at least three reads (deviating from Sommer et al. (2013)).

Second, the remaining variants above the 0.2% cut-off threshold in each amplicon were scored as follows: If the “=1bp” and “>1bp” variants were present in the amplicon replicate, they were scored as alleles. However, if a “>1bp” variant was present in other amplicons within the family group albeit not in replicate, it was nevertheless scored as allele. Chimeric variants were scored as allele if it was present as a non-chimeric sequence in amplicon replicate.

The two latter steps are however deviating slightly from the protocol in Sommer et al. (2013), in which “>2bp variants” are scored if present in other amplicons across the whole dataset while not in the replicate. We changed the original “>2bp” intra-amplicon evaluation category to “>1bp” according to the recommendations in Sommer et al. (2013), due to the expectancy of high complexity in bluethroat MHCII. Further, the “>1bp variants” were compared against the family group and not across the whole data set, as this will ensure increased applicability if used on datasets of different sizes. This treatment of “>1bp variants” will affect the observed “errors in pedigree”, calculated as the percentage of alleles in offspring not found in any of its parents, which is thus not directly comparable between the two genotyping pipelines. Lastly, the “unclassified variants” and “putative alleles” categories from Sommer et al. (2013) were collapsed to “Alleles”, due to the presence of high frequency artefacts.

Dealing with individuals with one failed replicate

Family information can prove particularly useful when genotyping complex markers, by assuming all true alleles within an offspring will be present in one or both of its parents. The genotype of the parents can thus be utilized to avoid loss of data in cases where an offspring has one amplicon without sufficient coverage for genotyping. Accordingly, we established a genotyping regime for offspring having one failed amplicon within a strategy, for whom parentage was known and the biological parents were included in the dataset. In these instances, the successful amplicon was analyzed following the protocol of the modified Sommer-pipeline until the artefact and allele calling (after step 10, main text Figure 3). The alleles of the parents were then used as a substitute for the failed amplicon in the subsequent steps: if a “=1bp” variant or a chimeric variant was scored as an allele in one of the parents, it was genotyped also for the focal offspring. The “>1 bp” variants were treated in the same manner as for every individual, and scored if found within any other family member.

## AmpliSAS

### Initial filtering:

MHCI-SI, MHCI-DI and MHCII-DI

Due to size limitations of input files in AmpliCLEAN, the initial quality and length filtering of the raw data was done using fastx_toolkit, as described for the modified pipeline from Sommer et al. (2013). The resulting output file was used as input for AmpliSAS twice, once for genotyping MHCI (using the MHCI-SI and MHCI-DI amplicons) and once for genotyping MHCII (using the MHCII-DI amplicons).

MHCII-SI

In order to treat the input files identically, the raw read file was quality and length trimmed using fastx_toolkit as described for MHCII-DI above. A fasta file containing the variants scored as alleles for MHCII-DI was used an input “Allele file” in order to name the variants identically.

MHCII-MiSeq

In order to obtain an input file of accepted size for the online version of AmpliSAS, AmpliMERGE and AmpliCLEAN were replaced by alternative approaches: the software FLASH was used to merge the sequence pairs from the paired end MiSeq run, while fastx_toolkit was used to filter the reads based on quality, with the same parameters as for the modified pipeline from Sommer et al. (2013). The merged, cleaned file was gzipped and used as input for AmpliSAS. A fasta file containing alleles from the AmpliSAS run of MHCII-SI and MHCII-DI was used as “Allele file”.

### Genotyping:

The online software suite AmpliSAS (Sebastian et al. 2016) was found at <http://evobiolab.biol.amu.edu.pl/amplisat/index.php?amplisas>. The respective input files were as stated above, and only variants scored in both amplicon replicates of an individual were called as alleles.

## References:

Anmarkrud, J., Johnsen, A., Bachmann, L., & Lifjeld, J. (2010). Ancestral polymorphism in exon 2 of bluethroat (*Luscinia svecica*) MHC class II B genes. *Journal of evolutionary biology,* 23, 1206-1217. doi: 10.1111/j.1420-9101.2010.01999.x

Babik, W., Taberlet, P., Ejsmond, M. J., & Radwan, J. (2009). New generation sequencers as a tool for genotyping of highly polymorphic multilocus MHC system. *Molecular Ecology Resources,* 9, 713-719. doi: 10.1111/j.1755-0998.2009.02622.x

Biedrzycka, A., Sebastian, A., Migalska, M., Westerdahl, H., & Radwan, J. (2017). Testing genotyping strategies for ultra-deep sequencing of a co-amplifying gene family: MHC class I in a passerine bird. *Molecular Ecology Resources,* 17, 642-655. doi: 10.1111/1755-0998.12612

Edgar, R. C., Haas, B. J., Clemente, J. C., Quince, C., & Knight, R. (2011). UCHIME improves sensitivity and speed of chimera detection. *Bioinformatics,* 27, 2194-2200. doi: 10.1093/bioinformatics/btr381

Galan, M., Guivier, E., Caraux, G., Charbonnel, N., & Cosson, J.-F. (2010). A 454 multiplex sequencing method for rapid and reliable genotyping of highly polymorphic genes in large-scale studies. *BMC genomics,* 11, 1. doi: 10.1186/1471-2164-11-296

Gohli, J., Anmarkrud, J. A., Johnsen, A., Kleven, O., Borge, T., & Lifjeld, J. T. (2013). Female promiscuity is positively associated with neutral and selected genetic diversity in passerine birds. *Evolution,* 67, 1406-1419. doi: 10.1111/evo.12045

Kumar, S., Stecher, G., & Tamura, K. (2016). MEGA7: Molecular Evolutionary Genetics Analysis version 7.0 for bigger datasets. *Molecular Biology and Evolution,* 33, 1870-1874. doi: 10.1093/molbev/msw054

Lighten, J., Oosterhout, C., & Bentzen, P. (2014). Critical review of NGS analyses for de novo genotyping multigene families. *Molecular Ecology,* 23, 3957-3972. doi: 10.1111/mec.12843

Magoč, T., & Salzberg, S. L. (2011). FLASH: fast length adjustment of short reads to improve genome assemblies. *Bioinformatics,* 27, 2957-2963. doi: 10.1093/bioinformatics/btr507

O'Connor, E., Strandh, M., Hasselquist, D., Nilsson, J. Å., & Westerdahl, H. (2016). The evolution of highly variable immunity genes across a passerine bird radiation. *Molecular Ecology*, 977-989. doi: 10.1111/mec.13530

Sebastian, A., Herdegen, M., Migalska, M., & Radwan, J. (2016). amplisas: a web server for multilocus genotyping using next-generation amplicon sequencing data. *Molecular Ecology Resources,* 16, 498-510. doi: 10.1111/1755-0998.12453

Sommer, S., Courtiol, A., & Mazzoni, C. J. (2013). MHC genotyping of non-model organisms using next-generation sequencing: a new methodology to deal with artefacts and allelic dropout. *BMC genomics,* 14, 1. doi: 10.1186/1471-2164-14-542

Stuglik, M. T., Radwan, J., & Babik, W. (2011). jMHC: software assistant for multilocus genotyping of gene families using next-generation amplicon sequencing. *Molecular Ecology Resources,* 11, 739-742. doi: 10.1111/j.1755-0998.2011.02997.x

Thompson, J. D., Higgins, D. G., & Gibson, T. J. (1994). CLUSTAL W: improving the sensitivity of progressive multiple sequence alignment through sequence weighting, position-specific gap penalties and weight matrix choice. *Nucleic Acids Research,* 22, 4673-4680. doi: 10.1093/nar/22.22.4673
