# Supplementary material for: Genotyping strategy matters when analyzing hypervariable major histocompatibility complex‐Experience from a passerine bird
Source: Ecol Evol. 2018 Jan 7;8(3):1680–92. doi: 10.1002/ece3.3757 (PMC5792522; doi:10.1002/ece3.3757)
Supplement: Supplementary file 14 [file ECE3-8-1680-s014.docx]

Appendix legends

Rekdal et al.: Genotyping strategy matters when analyzing hypervariable MHC

#### Appendix S1: Bluethroat individuals

Overview over the individuals studied. The accession numbers refer to DNA Bank accession numbers at the Natural History Museum of Oslo, Norway.

#### Appendix S2: Terms

Explanation of central terms as they are used in this study.

#### Appendix S3: Primer sequences

The sequences and lengths of the primers used in this study.

#### Appendix S4: Primer binding sites

The binding sites of the MHCI and MHCII primers. Modified figures from Alcaide et al. (2013) and Canal et al. (2010).

#### Appendix S5: Methods: sequencing details

Details of the amplification and sequencing of MHC class I exon 3 and MHC class II exon.

#### Appendix S6: Methods: allele calling details

Details of the allele calling of MHC class I exon 3 and MHC class II exon 2, based on the pipeline developed by Sommer et al. (2013) and the software AmpliSAS (Sebastian et al. 2016).

#### Appendix S7: Genotypes, MHCIe3

Genotypes for the eight individuals for MHC class I exon 3.

#### Appendix S8: Sequence logo, MHCIe3

Sequence logo created using the online tool WebLogo (<http://weblogo.berkeley.edu/logo.cgi>; Crooks et al. (2004)) for MHCI. Unique MHCI alleles identified among all strategies were aligned to previously described bluethroat MHCI alleles (GenBank accession number KU169737-KU169747; O'Connor et al. (2016)) by ClustalW (Thompson et al. 1994) in MEGA7 (Kumar et al. 2016), and translated correspondingly. Antigen-binding sites, as indicated as sites where d_N_/d_S_ > 1 in passerine MHCI exon 3 sequences by Balakrishnan et al. (2010), are marked with *. One sequence (LuSv_MHCIe3_SR_015) was omitted due to ambiguity in the placement of a deletion when aligned to the other alleles in MEGA.

#### Appendix S9: Network MHCIe3

A minimum spanning network (Bandelt et al. 1999) of the unique MHCI alleles identified in this study, made through the online software PopArt (<http://popart.otago.ac.nz>). The circles represent unique alleles found among the strategies, and their sizes are corresponding to the number of strategies they are found within. Additionally, they are color-coded according to the scheme in the lower right corner, implying in which strategies the alleles are found. The numbers are representing substitutions between alleles.

#### Appendix S10: Genotypes, MHCIIβe2

Genotypes for the eight individuals for MHC class II exon 2.

#### Appendix S11: Sequence logo, MHCIIβe2

Sequence logo created using the online tool WebLogo (<http://weblogo.berkeley.edu/logo.cgi>; Crooks et al. (2004)) for MHCII. Unique MHCII alleles identified among all strategies were aligned to previously described bluethroat MHCII alleles (GenBank accession number HQ539575-HQ539614; Gohli et al. (2013)) by ClustalW (Thompson et al. 1994) in MEGA7 (Kumar et al. 2016), and translated correspondingly. Antigen-binding sites, as indicated as sites where d_N_/d_S_ > 1 in passerine MHCII exon 2 sequences by Balakrishnan et al. (2010), are marked with *.

#### Appendix S12: Network MHCIIβe2

A minimum spanning network (Bandelt et al. 1999) of the unique MHCII alleles identified in this study, made through the online software PopArt (<http://popart.otago.ac.nz>). The circles represent unique alleles found among the strategies, and their sizes are corresponding to the number of strategies they are found within. Additionally, they are color-coded according to the scheme in the lower right corner, implying in which strategies the alleles are found. The numbers are representing substitutions between alleles.

#### Appendix S13: Venn diagram MHCIIβe2

Venn diagram showing the number of unique MHC II alleles found within each strategy, and the number of overlapping alleles among strategies. Made in R (version 3.2.5, R Core Team 2016), using the package VennDiagram (Chen & Boutros 2011).

## References:

Alcaide, M., Liu, M., & Edwards, S. V. (2013). Major histocompatibility complex class I evolution in songbirds: universal primers, rapid evolution and base compositional shifts in exon 3. *PeerJ,* 1, e86. doi: 10.7717/peerj.86

Balakrishnan, C. N., Ekblom, R., Völker, M., Westerdahl, H., Godinez, R., Kotkiewicz, H., . . . Warren, W. C. (2010). Gene duplication and fragmentation in the zebra finch major histocompatibility complex. *Bmc Biology,* 8, 1.

Bandelt, H. J., Forster, P., & Röhl, A. (1999). Median-joining networks for inferring intraspecific phylogenies. *Molecular Biology and Evolution,* 16, 37-48.

Canal, D., Alcaide, M., Anmarkrud, J. A., & Potti, J. (2010). Towards the simplification of MHC typing protocols: targeting classical MHC class II genes in a passerine, the pied flycatcher *Ficedula hypoleuca*. *BMC research notes,* 3, 236. doi: 10.1186/1756-0500-3-236

Chen, H., & Boutros, P. C. (2011). VennDiagram: a package for the generation of highly-customizable Venn and Euler diagrams in R. *BMC bioinformatics,* 12, 35.

Crooks, G. E., Hon, G., Chandonia, J.-M., & Brenner, S. E. (2004). WebLogo: a sequence logo generator. *Genome Research,* 14, 1188-1190.

Gohli, J., Anmarkrud, J. A., Johnsen, A., Kleven, O., Borge, T., & Lifjeld, J. T. (2013). Female promiscuity is positively associated with neutral and selected genetic diversity in passerine birds. *Evolution,* 67, 1406-1419. doi: 10.1111/evo.12045

Kumar, S., Stecher, G., & Tamura, K. (2016). MEGA7: Molecular Evolutionary Genetics Analysis version 7.0 for bigger datasets. *Molecular Biology and Evolution,* 33, 1870-1874. doi: 10.1093/molbev/msw054

O'Connor, E., Strandh, M., Hasselquist, D., Nilsson, J. Å., & Westerdahl, H. (2016). The evolution of highly variable immunity genes across a passerine bird radiation. *Molecular Ecology*, 977-989. doi: 10.1111/mec.13530

Sebastian, A., Herdegen, M., Migalska, M., & Radwan, J. (2016). amplisas: a web server for multilocus genotyping using next-generation amplicon sequencing data. *Molecular Ecology Resources,* 16, 498-510. doi: 10.1111/1755-0998.12453

Sommer, S., Courtiol, A., & Mazzoni, C. J. (2013). MHC genotyping of non-model organisms using next-generation sequencing: a new methodology to deal with artefacts and allelic dropout. *BMC genomics,* 14, 1. doi: 10.1186/1471-2164-14-542

Team, R. C. (2016). R: A language and environment for statistical computing. R Foundation for Statistical Computing, Vienna, Austria. Retrieved from https://www.R-project.org. ISBN: 3-900051-07-0

Thompson, J. D., Higgins, D. G., & Gibson, T. J. (1994). CLUSTAL W: improving the sensitivity of progressive multiple sequence alignment through sequence weighting, position-specific gap penalties and weight matrix choice. *Nucleic Acids Research,* 22, 4673-4680. doi: 10.1093/nar/22.22.4673
